# Plasma glutamine after acute or elective admission on the ICU

**DOI:** 10.1186/cc14482

**Published:** 2015-03-16

**Authors:** H Buter, M Koopmans

**Affiliations:** 1 MCL, Leeuwarden, the Netherlands

## Introduction

Low plasma glutamine concentrations are associated with unfavourable outcome at acute ICU admission. We questioned whether there is a difference in plasma glutamine level after acute or elective ICU admission.

## Methods

We performed a single-centre prospective observational study in a 22-bed mixed ICU. Exclusion criteria were age <18 years and total parental nutrition at admission. Patients were divided into two groups: elective surgery and acute admissions. Blood samples were taken at ICU admission and daily at 6.00 a.m. Glutamine levels were measured using the Bioprofile 100 plus analyser (Nova Biomedical UK, Cheshire, UK). A Mann-Whitney U test was used to detect differences between groups and a Bonferroni method to correct for multiple comparisons.

## Results

We included 88 patients after elective surgery (76 cardiac and 12 general surgery) and 90 patients after acute admission (27 sepsis, 17 acute surgery, two trauma and 44 medical). Baseline characteristics are presented in Table 1. Plasma glutamine levels at admission were significantly lower in acute patients compared with elective surgery, 0.25 mmol/l (IQR 0.09 to 0.37) versus 0.43 mol/l (IQR 0.33 to 0.55) (*P *< 0.001). There appeared to be a significant correlation between the APACHE IV score and glutamine levels (*R *= 0.52, *P *< 0.001). Moreover, in a backward linear regression analysis this correlation was independently associated with APACHE IV scores and the presence of infection, but not with the type of admission.

**Table 1:** 

	Elective	Acute
Age (years)	68 ± 10	59 ± 17^*^
APACHE IV	46 ± 14	67 ± 29^*^
LOS ICU	2 (2 to 2)	4 (2 to 7)^*^
Survival (*n*)	88	85^*^

Mean ± SD or median (IQR). ^*^P < 0.01.

## Conclusion

Plasma glutamine levels were significantly lower after acute admission compared with elective surgery. In both groups a considerable amount of patients had decreased glutamine levels, but this was not independently associated with the type of admission. In contrast to previous studies we found that glutamine levels were determined by severity of illness and the presence of an infection.

